# Mortality in Sepsis and its relationship with Gender

**DOI:** 10.12669/pjms.315.6925

**Published:** 2015

**Authors:** Nosheen Nasir, Bushra Jamil, Shahla Siddiqui, Najeeha Talat, Fauzia A. Khan, Rabia Hussain

**Affiliations:** 1 Nosheen Nasir, MRCP (UK), FCPS (Pakistan), Department of Medicine, Aga Khan University, P.O Box 3500, Stadium Road, Karachi 74800, Pakistan; 2 Bushra Jamil, FRCP (UK), Department of Medicine, Aga Khan University, P.O Box 3500, Stadium Road, Karachi 74800, Pakistan; 3 Shahla Siddiqui, FCCM, Diplomate American Board of Anesthesiology, Khoo Teck Puat Hospital, 90 Yishun central, Singapore 79688; 4 Najeeha Talat, PhD, Department of Paediatrics & Child Health and Department of Biological & Biomedical Sciences, Aga Khan University, P.O Box 3500, Stadium Road, Karachi 74800, Pakistan; 5 Fauzia A. Khan, FCPS, Department of Anaesthesia, Aga Khan University, P.O Box 3500, Stadium Road, Karachi 74800, Pakistan; 6 Rabia Hussain, PhD, FRCPath (UK), Department of Pathology and Microbiology, Aga Khan University, P.O Box 3500, Stadium Road, Karachi 74800, Pakistan

**Keywords:** Sepsis, Mortality, Gender, Cytokines, Interleukin-10, Interleukin-6, TNFα

## Abstract

**Background and Objective:**

Sepsis remains a leading cause of death across the world, carrying a mortality rate of 20–50%. Women have been reported to be less likely to suffer from sepsis and to have a lower risk of mortality from sepsis compared to men. The objective of this study was to determine the relationship between gender and mortality in sepsis, and compare cytokine profiles of male and female patients.

**Methods:**

This was a prospective case series on 97 patients admitted with sepsis. Clinical and microbiological data was gathered, blood samples were collected for cytokine (IL-10, IL-6 and TNFα) levels and patients were followed up for clinical outcome.

**Results:**

There were 54% males and 46% females, with no significant difference of age or comorbids between genders. Respiratory tract infection was the commonest source of sepsis, and was more common in females (60%) compared to males (39%) (p=0.034). Males had a higher mortality (p=0.048, RR 1.73) and plasma IL-6 level(p=0.040) compared to females. Mean IL-6 plasma level was significantly (p<0.01) higher in patients who died vs. who recovered.

**Conclusion:**

Our study shows that males with sepsis have a 70% greater mortality rate, and mortality is associated with a higher IL-6 plasma level.

## INTRODUCTION

Sepsis has been a major cause of mortality and morbidity worldwide. It is the leading cause of death overall and is the most common cause of shock in the United States.^1^,^2^ Despite recent advances in diagnosis and management, mortality from sepsis remains high, ranging from 15% in patients with sepsis to 40-50% in patients with septic shock with multi-organ dysfunction syndrome (MODS).^3^ It has been observed that hormonal differences may play a role in development of chronic autoimmune and inflammatory diseases such as multiple sclerosis, lupus, or rheumatoid arthritis in women.^4^ Studies have shown that the immune response to infection as well as the incidence of sepsis differs between sexes.^5^ Data from animal studies suggests that females have advantageous immunologic responses during infections.^6^ Epidemiologic studies consistently report higher sepsis incidence in males.^7^,^8^ However, the influence of gender on severe infections is still highly controversial and although animal studies have indicated a survival advantage for females,^6^ it seems to be contradictory to human clinical data on sepsis related mortality. Eachempati et al.^9^ has highlighted that female gender as an independent predictor of increased mortality in patients with documented infection in a surgical intensive care unit (ICU). Hence the data seen in sepsis patients with respect to differences in outcomes in relation to gender has so far has been equivocal.

This study is part of a larger study to evaluate the characteristics of sepsis patients and its purpose is to study the relationship between gender and mortality due to sepsis and to identify differences if any between the cytokine levels specifically IL-6, IL-10 and TNF alpha between the genders.

## METHODS

We conducted a prospective observational study at Aga Khan University Hospital, Karachi, between 2005 and 2006. The study was approved by the Ethics Review Committee of Aga Khan University, and we recruited patients from the hospital clinical areas after obtaining informed consent. A total of 100 patients of age >18 years, admitted with sepsis and septic shock as defined by Society of Critical Care Medicine SCCM were included. Sepsis was defined as presence of infection along with presence of two out of following four parameters: body temperature higher than 38°C or lower than 36°C, heart rate higher than 90/min, hyperventilation evidenced by respiratory rate higher than 20/min or PaCO_2_ lower than 32 mmHg and white blood cell count higher than 12,000 cells/μl or lower than 4,000/μl. Patients with moderate, severe or life threatening infection s were included. Life threatening infection was defined as presence of septic shock, and was severe infection if patient had severe sepsis with presence of lactic acidosis and hypotension of SBP <90 or SBP Drop ≥ 40 mm Hg of normal which was responding to IV fluids and moderately severe infection which fulfilled the criteria of sepsis and required hospital admission and use of intravenous antimicrobial therapy. Patients with systemic inflammatory response syndrome (SIRS) but not having any infection were excluded. Clinical and microbiological data was gathered on a proforma. Variables studied included age, gender, co-morbids, site of infection, microbial pathogen isolated and outcome measures included in-hospital mortality as opposed to recovery and discharge. Types of infection studied included lower respiratory tract infection including community acquired pneumonia and health care associated pneumonia, urinary tract infections including catheter associated UTI, intra-abdominal infections including abscesses and peritonitis, skin and soft tissue infections, bloodstream infections including central line associated bloodstream infections, central nervous system infections, genitourinary infections and septic arthritis and osteomyelitis patients were followed up for clinical outcome.

### Blood sample collection

Blood samples were collected in EDTA tube. The plasma was separated by centrifugation @ 2000rpm for 10mins. Clear supernatant was collected and stored at -80°C until use.

### Cytokine ELISA

Cytokine (IL-10, IL-6 and TNFα) levels were measured using ELISA. Briefly, Immulon 4 plates were coated with capture antibody specific for the human TNFα, IL6, and IL10 and incubated overnight at 4°C. Non-specific binding sites were blocked with 3% bovine serum albumin (BSA) in phosphate buffer saline (PBS) for 2 hour at room temp. Neat Plasma samples were added in respective wells according to plate template. A dose response curve starting from 1000-7.8 pg/ml for every cytokine was run simultaneously with samples. Plates were further incubated overnight at 4°C. Next day, the captured cytokines was then incubated with biotinylated probing antibodies specific for the cytokine to be detected. The revealing probe was avidin bound to horseradish peroxidase (HRP) were used as conjugate. The plates were washed three times in between each incubation, with PBS containing 0.05% Tween-20 to remove any unbound protein. The plates were finally developed for color reaction using OPD tablets @ 2mg/ml in Na perborate buffer. The intensity of color reaction is proportional to amount of cytokine present in serum. All plates were read on Biorad at 450 nm.

### Statistical analysis

Data was analyzed on SPSS. Numerical variables were expressed as mean ± standard error of the mean. Chi square or Fisher exact test was used for group comparisons of categorical variables and Mann Whitney test U for continuous variables. P-value < 0.05 was considered significant.

## RESULTS

Out of 100 patients with sepsis, 97 were included for analysis, exclusions being due to incomplete information. 54% were male and 46% female. The age distribution was similar in both the genders with mean age being 54±2 years in males and 50±2 in females (p=0.30). Diabetes mellitus was the leading co-morbid present in 21 males as opposed to 18 females, followed by hypertension in 22 males and females respectively. There were no significant differences among gender for co-morbids (Table-I). Respiratory tract infection was the most common source of sepsis, and was significantly more common in females compared to males (60% vs. 39% respectively, p=0.034). Urinary tract infection was the next common source but no gender difference was observed (Table-I). Gram negative organisms accounted for about 60% of the infections in both genders and E.coli was identified as the most frequent pathogen cultured from various sites in both the sexes (22%). Septic shock was present in 16 males and 13 females.

**Table-I:** Comparison of patient characteristics between genders (n,%).

	Male (n=52)	Female (n=45)	P-value
Age in years (mean±SD)	54±17	50±16	0.300
Sepsis on admission	36 (69%)	32 (71%)	0.840
Septic shock on admission	16 (31%)	13 (29%)	0.840
*Co-morbids*			
Diabetes mellitus	21 (40%)	18 (40%)	0.969
Hypertension	22 (42%)	22 (49%)	0.516
Ischemic heart disease	11 (21%)	11 (24%)	0.670
Chronic renal disease	12 (23%)	12 (27%)	0.683
Chronic liver disease	5 (10%)	1 (2%)	0.211
*Type of infection*			
Lower respiratory tract Infection	20 (38%)	27 (60%)	0.034
Urinary tract infection including CAUTI	17 (33%)	20 (44%)	0.235
Bloodstream infection including CLABSI	11 (21%)	14 (31%)	0.264
Intra-abdominal infection	5 (10%)	0 (0%)	0.033
Skin & soft tissue Infection	4 (8%)	4 (9%)	0.831
CNS infection	2 (4%)	4 (9%)	0.304
Others	9 (17%)	6 (13%)	0.589
*Microbiological Data*			
Culture Positive	26 (50%)	23 (51%)	0.913
Gram positive organisms isolated	8 (15%)	6 (13%)	0.774
Gram negative organisms isolated	15 (29%)	14 (31%)	0.808
Polymicrobial growth	3 (6%)	3 (7%)	0.855

**Fig.1:**
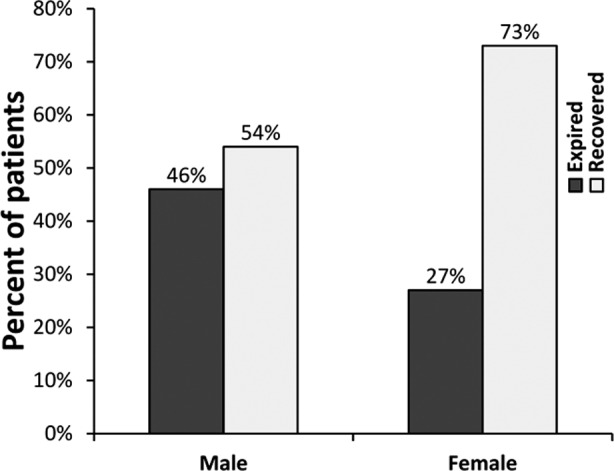
Bar chart comparing proportion of male and female patients expired and recovered. Difference was significant (p=0.048, Chi square test).

**Fig.2:**
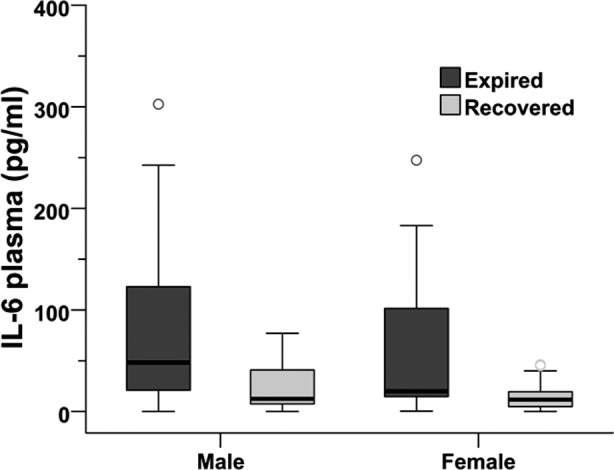
Box plot showing median (50%), 25% and 75% quartiles of IL6 (pg/ml) values in male and females patients in both expired (n=36) and recovered (n=61) categories. The IL6 levels were determined using an in-house sandwich ELISA assay in serum samples (dilution factor). All samples were run in duplicate and mean values of individual samples were entered in SPSS 13.0 for analysis. Difference of mean values between expired and recovered categories within the genders was significant at p<0.05, Mann Whitey U test.

As for outcome, males had a significantly greater mortality compared to females (46% vs. 27%, p=0.048) (Fig.1), with relative risk (RR) of 1.73 (0.98 <RR< 3.05). On categorizing patients into two groups based on age >50 years and < 50 years, no significant difference was observed in mortality. Mean plasma level of TNFα was 193.15±61.92 pg/ml in males and 91.44±20.80pg/ml in females but this was not statistically significant (p=0.972). Similar to TNFα, mean plasma level of IL-10 was also not significantly different (p=0.940) between genders, i.e. 188.26±51.47 pg/ml in males and 164.29 ±34.25pg/ml in females. However, mean plasma level of IL-6 was 60.72±13.41 pg/ml in males and 28.06±7.16 pg/ml in females which was two-fold higher in males compared to females (p=0.025, Mann Whitney U test). Notably, mean IL-6 plasma level was also significantly (P<0.01) higher in patients who died vs. who recovered (Fig.2).

As gender and IL-6 levels had significant relationship with mortality, to determine possible interdependency of these variables with comorbids and site of infection, logistic regression analysis was performed. The results (Table-III) showed that gender is related to mortality independent of other factors. Moreover, urinary and respiratory tract infections, and IL-6 levels were independently associated with mortality. Comorbids (diabetes and ischaemic heart disease) did not have an independent relation to mortality.

**Table-II:** Comparison of microbiological and cytokine data and outcome between genders.

	Male (n=52)	Female (n=45)	P-value
Microbiological Data (n,%)			
Culture Positive	26 (50%)	23 (51%)	0.913
Gram positive organisms isolated	8 (15%)	6 (13%)	0.774
Gram negative organisms isolated	15 (29%)	14 (31%)	0.808
Polymicrobial growth	3 (6%)	3 (7%)	0.855
Cytokines (pg/ml; mean±SD)			
TNFα	193.15±61.92	91.44±20.80	0.972
Interleukin 6	60.72±13.41	28.06±7.16	0.025
Interleukin 10	188.26±51.47	164.29±34.25	0.940
Outcome (n,%)			
Expired	24 (46.2%)	12 (26.7%)	0.048
Recovered	28 (53.8%)	33 (73.3%)	
			

**Table-III:** Multivariate analysis using logistic regression with mortality as dependent variable. Gender, two sites of infection (respiratory and urinary tracts) and plasma IL-6 were independently associated with mortality.

Variable	P-value
Gender	0.046
Age group	0.254
Diabetes mellitus	0.691
Ischaemic heart disease	0.367
Respiratory tract infection	0.006
Urinary tract infection	0.029
Plasma IL-6 level	0.016

## DISCUSSION

Our study has shown greater mortality among males as compared to females with sepsis. This phenomena had been observed in animal studies where females have been known to have survival advantage in terms of both immunologic as well as cardiovascular responses.^6^,^10^ However, most clinical studies have failed to show consistent differences in sepsis outcomes in relation with gender. Eachempati et al.^9^ has highlighted that female gender as an independent predictor of increased mortality in patients with documented infection in a surgical intensive care unit (ICU). A study on patients admitted in intensive care showed slight higher odds of mortality in females versus males (OR 1.11) in the subgroup with severe sepsis.^11^ They performed multivariate analysis to show that gender had independent effect on mortality, analysis showed higher mortality in females with severe sepsis. This is in contrast to our study, where males had higher mortality. The difference may be due to differences in the patient population. Similarly, a study from Germany concluded that there were no differences in patients’ outcome related to gender aspect in mainly surgical ICUs. However, for patients with sepsis, an increase of mortality is related to the female sex.^12^ On the other hand Wichmann et al.^13^ concluded from his study that a significantly smaller number of female patients requiring intensive care as well as a significantly lower incidence of severe sepsis/septic shock in female intensive care patients. Despite these findings mortality from severe sepsis/septic shock was not affected by gender. This is in contrast to our finding where male patients were at a greater risk for dying from sepsis. These differences may be due to surgical ICU setting which was studied by Nachtigall et al.^12^ and Wichmann et al.^13^ as opposed to our ICU where there is representation from both surgical and medical patients. However, one of our limitations is a small sample size and perhaps a large sample is needed for better evaluation of the difference observed. One of the probable reasons is the accessibility and priority of males to be admitted in hospital compared to females.

Moreover role of cytokines has been extensively studied in order to gain better insight into the processes that influence outcome in sepsis. Multiple organ dysfunction is due to a severe inflammatory reaction resulting from systemic cytokine release.^14^ The pro-inflammatory reaction is mediated by tumor necrosis factor (TNF-a), interleukin (IL) 1 and IL-6. The body also mounts an immediate anti-inflammatory response largely mediated by IL-10.^15^ In a prospective study from Germany, gender differences in patients with surgical sepsis were evaluated in terms of survival, sex hormones, and proinflammatory as well as anti-inflammatory mediators. The study demonstrated a significantly better prognosis for women, which may be related to increased levels of anti-inflammatory mediator IL-10.^16^ Although our study also showed better outcome in women but there was no statistically significant difference in IL-10 levels in either group with a trend towards higher level in men as compared to women. Schroder et al.^16^ also observed sustained elevation of IL-6 level in patients with multiple organ dysfunction and a high mortality rate but there was no difference between women and men whereas bioactivity of TNF-a increased continuously in men as opposed to low levels in women. This is in contrast to our finding where IL-6 was associated with overall greater mortality and was significantly higher in men. This could reflect differences in population being studied. In our study, women had; significantly higher frequency of respiratory tract infection. However a multicenter study from 3 different countries has shown greater incidence of respiratory infections in men as compared to women.^11^ These findings may need to be better evaluated in a study with large sample size.

As to the possible reasons why our study showed a higher mortality rate in men compared to women, our data raises several possibilities: 1) men had higher level of IL-6 in our study and other studies have shown that high IL-6 is related to higher mortality,^16^ 2) there may be differences in accessibility to intensive care in our socio-cultural set up where males may get preference when they are more sick, 3) nature of patients in our ICU, which is a combined medical and surgical ICU, may be different from other studies.

A limitation of our study is that we were unable to use predictive scores such as APACHE. Such scores are generally applied in the ICU setting while, many of our patients were not admitted in ICU. Several parameters used for such scores were not available in all our patients, e.g. arterial blood gases, bilirubin, hourly urine output. Moreover, the relatively small sample size is another limitation; a larger sample size may have permitted meaningful subgroup analyses.

## CONCLUSION

We have found that males with sepsis have a 70% greater mortality rate as compared to females. This higher mortality appears to be related to differences in respiratory tract infection rate and IL-6 plasma levels, between the genders.
